# Pre-operative CT scan measurements for predicting complications in patients undergoing complex ventral hernia repair using the component separation technique

**DOI:** 10.1007/s10029-019-01899-8

**Published:** 2019-03-07

**Authors:** H. Winters, L. Knaapen, O. R. Buyne, S. Hummelink, D. J. O. Ulrich, H. van Goor, E. van Geffen, N. J. Slater

**Affiliations:** 1 Department of Plastic and Reconstructive Surgery, Radboud University Medical Center, PO Box 9101, 6500 HB Nijmegen, The Netherlands; 2 Department of Surgery, Radboud University Medical Center, PO Box 9101, 6500 HB Nijmegen, The Netherlands; 3 Department of Surgery, Jeroen Bosch Hospital, PO Box 90153, 5200 ME ’s Hertogenbosch, The Netherlands

**Keywords:** Complex ventral hernia, Component separation technique, Ramirez, BMI, Fat volume, Body morphometrics, Reherniation

## Abstract

**Background:**

The component separation technique (CST) is considered an excellent technique for complex ventral hernia repair. However, postoperative infectious complications and reherniation rates are significant. Risk factor analysis for postoperative complication and reherniation has focused mostly on patient history and co-morbidity and shows equivocal results. The use of abdominal morphometrics derived from CT scans to assist in risk assessment seems promising. The aim of this study is to determine the predictability of reherniation and surgical site infections (SSI) using pre-operative CT measurements.

**Methods:**

Electronic patient records were searched for patients who underwent CST between 2000 and 2013 and had a pre-operative CT scan available. Visceral fat volume (VFV), subcutaneous fat volume (SFV), loss of domain (LOD), rectus thickness and width (RT, RW), abdominal volume, hernia sac volume, total fat volume (TFV), sagittal distance (SD) and waist circumference (WC) were measured or calculated. Relevant variables were entered in multivariate regression analysis to determine their effect on reherniation and SSI as separate outcomes.

**Results:**

Sixty-five patients were included. VFV (*p* = 0.025, OR = 1.65) was a significant predictor regarding reherniation. Hernia sac volume (*p* = 0.020, OR = 2.10) and SFV per 1000 cm^3^ (*p* = 0.034, OR = 0.26) were significant predictors of SSI.

**Conclusion:**

Visceral fat volume, subcutaneous fat volume and hernia sac volume derived from CT scan measurements may be used to predict reherniation and SSI in patients undergoing complex ventral hernia repair using CST. These findings may aid in optimizing patient-tailored preoperative risk assessment.

## Introduction

Complex ventral hernia repair remains a very challenging domain for general and reconstructive surgeons. Synthetic mesh bridging is used for small ventral hernias but coincides with a high reherniation rate and bulging of the abdomen when used for large and more complex defects and should be avoided in these cases [1–3]. The component separation technique (CST) is considered an excellent technique to close large complex defects as this procedure reestablishes the abdominal wall integrity. In this procedure, the external oblique muscles are released to facilitate the sliding of these myofascial flaps to allow re-approximation of the rectus abdominis [4]. Additional mobilization is achieved by release of the posterior rectus sheath from the rectus muscle. Studies with reliable and lengthy follow-up show high reherniation rates up to 37.7% after large ventral hernia repair after CST without mesh [5] While retrospective studies demonstrate a lower reherniation risk when mesh reinforcement is used, there are no studies comparing reherniation risk head to head with or without use of mesh [6, 7]. In the literature, risk factor analysis for postoperative complication and reherniation has focused almost exclusively on patient history and co-morbidity (e.g., obesity, smoking, previous reherniation). While surgeons heavily rely on physical exam and CT scans, CT scan measurements have not been extensively studied to predict adverse outcomes [8, 9].

Computed tomography (CT) scans are more and more routinely performed during patient workup for adequate preoperative planning of large ventral hernias. While the scans can be used to determine hernia characteristics, more abdominal characteristics can be measured using a dedicated workstation, including the visceral and subcutaneous fat components of the abdomen. It is known that for predicting complications in colorectal surgery, visceral fat volume is more accurate in predicting the risk to the development of an incisional hernia than BMI [10]. Since BMI does not accurately reflect visceral fat mass, it is likely that these results can be translated to other forms of surgery including complex ventral hernia repair.

The aim of the current study is to explore the use of CT scan-derived body morphometrics [visceral fat volume (VFV), subcutaneous fat volume (SFV), total fat volume (TFV) and loss of domain (LOD)] to predict reherniation and SSI in patients undergoing complex ventral hernia repair using the CST. This may allow for a more reliable quantitative risk analysis leading to better-informed decisions regarding efficacy and safety of surgery and inform patients in a patient-tailored fashion.

## Patients and methods

### Study population and surgical procedure

The study was performed following a retrospective cohort design. Electronic patient records were searched for patients who met the inclusion criteria, after which a chart review (using a pre-defined case report form) was performed. Adult patients (18–75 years of age at time of operation) who underwent complex ventral hernia repair using the CST (with or without the use of mesh reinforcement) between 2000 and 2013 and who had a preoperative CT scan were eligible for inclusion. Patients were excluded if the CT scan was performed earlier than 6 months prior to surgery to minimize the influence of changes in weight, or if the scan did not cover the full abdomen. Patient demographics, operation data, the occurrence of reherniation, SSI within 30 days and follow-up information were extracted (Table 1). SSI was defined according to the centers for disease control and prevention (CDC) definitions for surgical site infection.

**Table 1:** Peri-operative patient characteristics

Peri-operative variables	*n* (%)
Patients	65 (100)
Male	49 (75.4)
BMI (kg/m^2^), median (range)	26.3 (20–37.2)
Obesity, BMI > 30	12 (18.5)
Age, median (range)	62 (23–78)
Mesh reinforcement	45 (69.2)
Vypro mesh	22 (33.1)
Proceed mesh	17 (25.4)
Ultrapro mesh	4 (6.2)
Prolene mesh	2 (3.1)
Sepra mesh	1 (1.5)
Operation duration, median (range)	206.5 (26–420)
Blood loss, median (range)	750 (150–2000)
Surgical site infection > 30 days	14 (21.5)
Reherniation	18 (27.7)
Follow-up, median (range)	14 (0–82)

CST was performed as previously described [5]. Briefly, uni- or bilateral release of the external oblique aponeurosis is performed to achieve medial translation of the rectus complex, with or without mobilization of the posterior rectus sheath for additional medialization. The aim is always to close the posterior fascia and also spare the peri-umbilical perforators if possible. A synthetic mesh was used only if there was no concurrent infection during reconstruction of the abdominal wall. If a mesh was used it was placed with an overlap of 5 cm on each side of the defect. In some patients with a non-contaminated wound, reconstruction without mesh reinforcement was used because of a concurrent trial that was being performed at the time. This study aimed to compare the results of mesh reinforcement in addition to CST and CST without mesh reinforcement. All procedures were performed exclusively by a group of four experienced abdominal wall surgeons.

### Computed tomography measurements

**Fig. 1:**
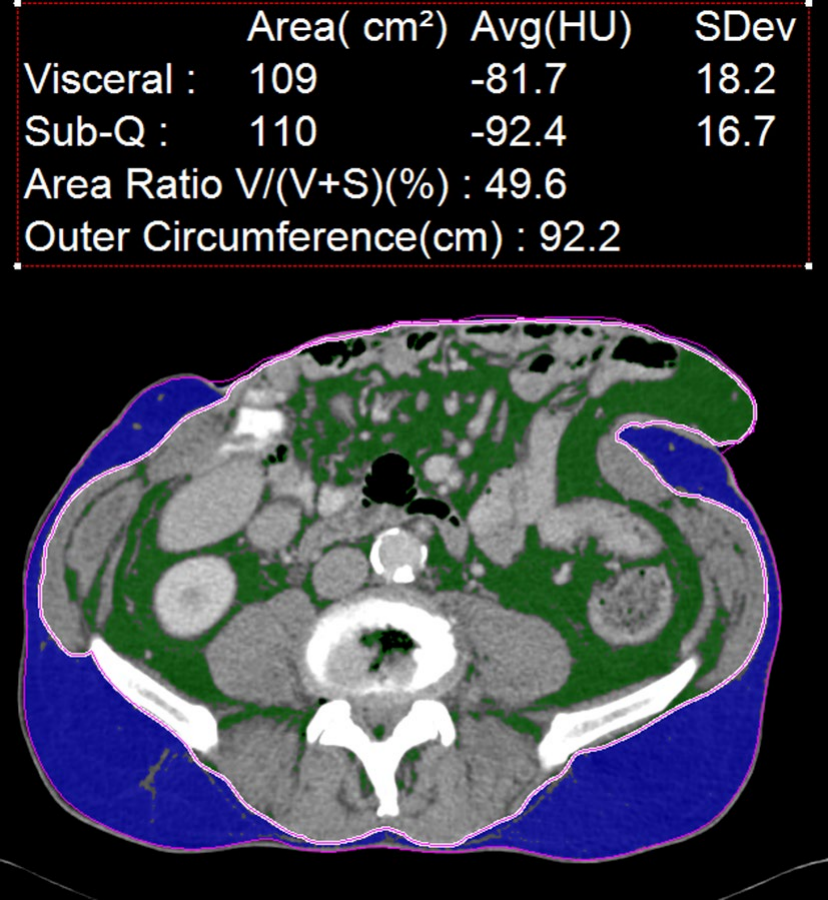
Quantification of the visceral and subcutaneous fat. The green and blue sections represent the visceral and subcutaneous fat, respectively. (Color figure online)

The CT scans were analyzed by the corresponding author (HW) using a dedicated workstation (Aquarius 3D Workstation, TeraRecon, San Mateo, CA, USA), which was able to separate intra- from extra-abdominal fat. Fat volumes were analyzed using a modified measurement protocol described by Rickles et al. [11]. The VFV, SFV and waist circumference (WC) were measured semi-automatically every 1.2 cm up to 12 cm cranially from the most cranial slice in which S1 was still visible. The range of Hounsfield units (HU) to determine adipose tissue was between − 150 and − 50. Measurements were manually corrected if the intra-abdominal area was not correctly demarcated from the extra-abdominal area (Fig. 1). All measurements were systematically re-evaluated by HW, NJS and SH and differences were solved by consensus.

**Fig. 2:**
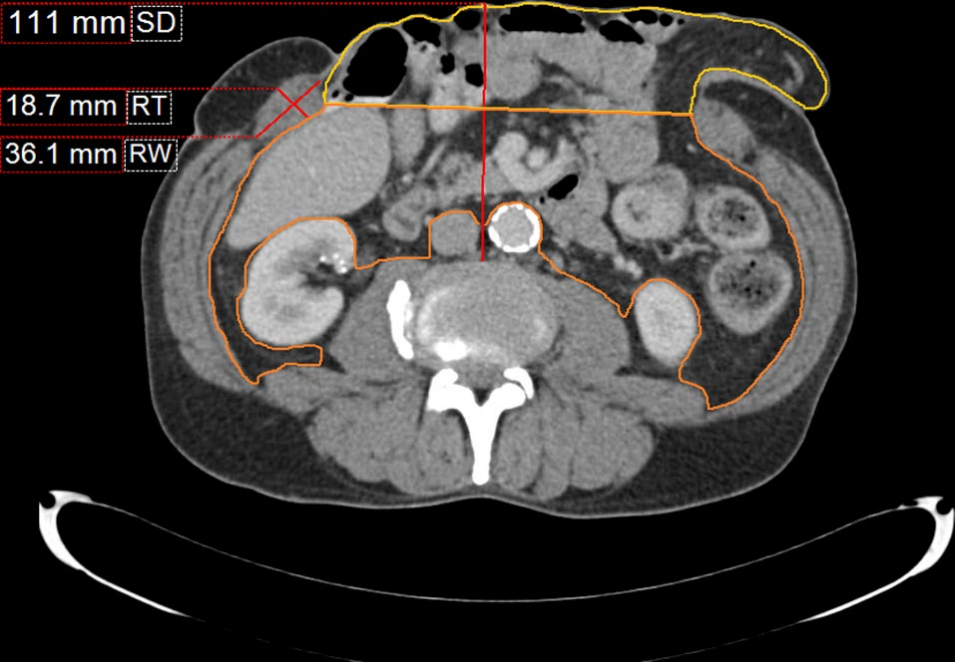
CT measurements. The orange outline represents the intra-abdominal area whereas the yellow outline represents the hernia sac. SD, RT and RW can be seen on the left. (Color figure online)

**Fig. 3:**
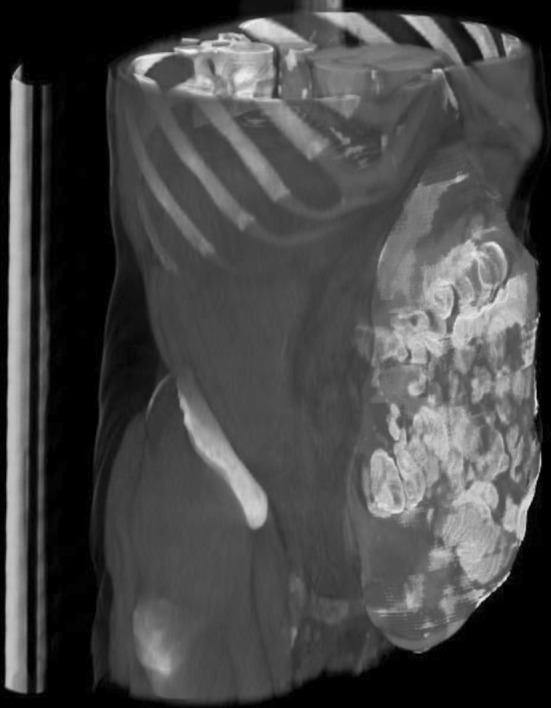
3D reconstruction of the abdomen. The colored part represents the hernia and the abdomen which were used for calculation of segmentation volumes of the abdomen and hernia sac. (Color figure online)

Rectus abdominis muscle thickness and width were measured at L2–4 level. Sagittal diameter (SD: the distance from the corpus vertebrae to the internal boundary of the abdominal wall) and WC were measured at the umbilical level (at the intervertebral disc between L3 and L4; Fig. 2). To determine LOD, a 3D reconstruction was made from the abdomen by tracing the abdomen and hernia sac from the pubic bone to the diaphragm using at least 20 manual measurements. The intermediate measurements were calculated by the software and then manually checked for accurate separation of the intra-abdominal and extra-abdominal area. If necessary, manual correction was performed for each slice of the scan. Abdominal volume, hernia sac volume and LOD were calculated based on these measurements (Fig. 3). Measurement results are presented in Table 2.

**Table 2:** Computer tomography measurements

Computer tomography measurements	Mean (range)
Rectus thickness (mm)	14.17 (7.09–51.90)
Rectus width (mm)	54.78 (20.20–133.13)
Abdominal volume (cm^3^)	8937 (3698–13,983)
Hernia sac volume (cm^3^)	658 (0–2600)
Loss of domain	6.82 (0.00–29.72)
Total fat (cm^2^)	379.86 (87.54–661.18)

	Male	Female
Subcutaneous fat (cm^2^)	165 (38.2–405)	226 (99.9–419)
Visceral fat (cm^2^)	217 (49.4–398)	143 (49.5–310)
Waist circumference (cm)	102 (83.8–120)	95.9 (81.8–114)
Sagittal distance^a^ (cm)	138 (77.5–200)	115 (73.8–166)

^a^The distance between the corpus vertebrae and the abdominal wall

### Statistical analysis

For all measured variables, a Cox regression analysis was performed with reherniation as outcome and a univariate binary logistic regression was performed with SSI as outcome. Variables were included in multivariate Cox regression with reherniation as outcome and binary logistic regression analysis with SSI as outcome if the *p* value was < 0.2 in univariate analysis. To prevent the inclusion of highly correlating variables in multivariate Cox and logistic regression analysis, the Pearson correlation coefficients between variables were determined. Out of CT scan measurements with a Pearson correlation coefficient > 0.7 the one with the lowest *p* value was entered in the multivariate analysis [12]. Variables with a *p* value < 0.05 were considered significant in multivariate analysis.

A scatter plot was created for VFV versus body mass index (BMI) to determine if BMI is a predictor of visceral fat. All analyses were performed with IBM SPSS Version 22 (IBM Corp., Armonk, N.Y.).

## Results

A total of 209 patients were operated for complex ventral hernia using CST between 2000 and 2013. 65 patients had a CT scan performed within 6 months before surgery and both the CT scans and the follow-up were performed at our hospital. Patient demographics and peri-operative variables are described in Tables 1and 2. Briefly, in our population, reherniation occurred in 18 patients (27.7%) and SSI occurred in 14 patients (21.5%). The average follow-up (defined as clinical examination in the out-patient clinic) was 14 months (range 0–82). Mesh reinforcement was used in 45 patients (69.2%). Mesh was placed in a sublay (73.1%), intraperitoneal (16.2%), onlay (7.6%) or inlay (1.5%) position. Missing variables are demonstrated in Table 3.

**Table 3:** Missing variable analyses

Variable	*n* (%)
BMI	4 (6.2)
Obese	4 (6.2)
OK duration	1 (1.5)
Blood loss	12 (18.5)
Size defect	3 (4.6)
Rectus thickness	4 (6.2)
Rectus with	6 (9.2)
Hernia volume	1 (1.5)
Abdominal volume	2 (3.1)
Loss of domain	3 (4.6)
Waist circumference	1 (1.5)
Visceral fat volume	2 (3.1)
Subcutaneous fat volume	3 (4.6)
Total fat volume	2 (3.1)

### Reherniation

The use of a mesh (*p* = 0.074, OR = 0.42), VFV (*p* = 0.029, OR = 1.67) and TFV (*p* = 0.139, OR = 1.27) were predictors for reherniation in univariate Cox regression (Table 4). Mesh reinforcement was a protective predictor of reherniation where an increase of VFV and TFV increased the risk of recurrence. VFV and TFV correlated with *r* = 0.81. Since VFV and TFV correlated > 0.7 and VFV had a lower *p* value, TFV was omitted from multivariate analysis. Therefore, VFV and the use of a mesh were entered into a multivariate Cox regression analysis with reherniation as outcome.

**Table 4:** Univariate risk analysis results

Variable	Univariate OR (95% CI), *p* value	
	Reherniation	Infection
Sex (female)	0.69 (0.23–2.10), 0.512	0.19 (0.22–1.54), 0.119*
BMI	1.08 (0.94–1.23), 0.240	0.92 (0.78–1.09), 0.325
Mesh	0.42 (0.16–1.09), 0.074*^,†^	1.83 (0.45–7.46), 0.397
SSI	0.61 (0.18–2.14), 0.443	–
Rectus thickness, per 10 mm	3.26 (0.42–25.24), 0.258	1.46 (0.66–3.20), 0.365
Rectus width, per 10 mm	1.23 (0.75–2.03), 0.419	1.13 (0.81–1.58), 0.455
Abdominal volume, per 500 cm^3^	1.42 (0.82–2.44), 0.213*	0.91 (0.71–1.19), 0.755
Hernia sac volume, per 500 cm^3^	1.13 (0.80–1.59), 0.480	1.41 (0.92–2.16), 0.119*
Loss of domain, per 5%	0.41 (0.16–1.09), 0.956	1.39 (0.93–1.75), 0.128*
Waist circumference, per 10 cm	1.23 (0.63–2.02), 0.538	0.96 (0.28–1.09), 0.090*
Sagittal distance^a^, per 10 cm	1.08 (0.91–1.27), 0.400	1.05 (0.86–1.28), 0.603
Visceral fat, per 1000 cm^3^	1.67 (1.05–2.64), 0.029*^,†^	0.72 (0.41–1.25), 0.246
Subcutaneous fat, per 1000 cm^3^	1.29 (0.81–2.09), 0.285	0.31 (0.12–0.81), 0.018*^,†^
Total fat, per 1000 cm^3^	1.27 (0.93–1.74), 0.139*	0.67 (0.44–1.03), 0.065*
Defect size, per cm^2^	0.99 (0.99–1.00), 0.798	1.00 (0.99–1.01), 0.910
Number of previous repairs	1.03 (0.76–1.42), 0.835	1.58 (0.94–2.67), 0.087*

	*B*	S.E.	Wald	*df*	Sig.	Exp(B)	95% CI for Exp(B)	
							Lower	Upper
Step 1^b^								
Sex	− 1.983	1.571	1.592	1	0.207	0.138	0.006	2.994
v_br_per_500	0.187	0.763	0.060	1	0.806	1.206	0.270	5.382
lod_per_5	0.521	0.676	.0595	1	0.440	1.684	0.448	6.331
sv_vol_per_1000	− 1.308	0.647	4.089	1	0.043	0.270	0.076	0.961
n_prev_repair	0.882	0.522	2.859	1	0.091	0.417	0.869	6.721
Constant	1.1527	1.584	0.929	1	0.335	4603		

^a^The distance between the corpus vertebrae and the abdominal wall

^b^Variable(s) entered on step 1: sex, v_br_per_500, lod_per_5, sv_vol_per_1000, n_prev_repair

*Significant during univariate analysis

^†^Significant during multivariate analysis

In the multivariate analysis, only VFV per 1000 cm^3^ (*p* = 0.025, OR = 1.65) was a significant predictor for reherniation where an increase in VFV increases the risk of recurrence. The use of mesh reinforcement (*p* = 0.062, OR = 0.15) was not statistically significant as a protective factor in this multivariate model.

### Surgical site infection

Significant predictors of SSI in univariate analysis were female sex (*p* = 0.119, OR = 0.19), hernia sac volume (*p* = 0.119, OR = 1.41), LOD (*p* = 0.128, OR = 1.39) SFV (*p* = 0.018, OR = 0.31), TFV (*p* = 0.065, OR = 0.67) and number of previous hernia repairs (*p* = 0.087, OR = 1.58). Hernia sac volume and LOD correlated with *r* = 0.94. Therefore, LOD was excluded from multivariate analysis. SFV and TFV correlated with *r* = 0.75. Therefore, TFV was excluded from multivariate analysis. In multivariate binary logistic regression analysis, hernia sac volume and SFV proved to be significant predictors (*p* = 0.020, OR = 2.10 and *p* = 0.034, OR = 0.26, respectively). Number of previous hernia repairs and sex were not significant (*p* = 0.089, OR = 2.24 and *p* = 0.252, OR = 0.239 respectively).

### Fat volume correlations

BMI and VFV were only weakly correlated (*r* = 0.45), whereas BMI was more strongly correlated to total fat and subcutaneous fat (*r* = 0.73 and *r* = 0.75, respectively).

## Discussion

In this study, we demonstrate that reherniation rate after hernia repair using the CST can be predicted by VFV measured on a pre-operative CT scan. For every 900 g (1.98 lbs) increase of visceral fat, the risk of reherniation almost doubled. In addition, hernia sac volume and SFV are predictors of SSI (OR = 2.05 and OR = 0.22, respectively). Interestingly, in our population, an increase of subcutaneous fat mass decreased the risk to develop SSI. These findings suggest that CT measurements are a valuable tool for pre-operative risk assessment in patients undergoing complex ventral hernia repair using the CST.

**Fig. 4:**
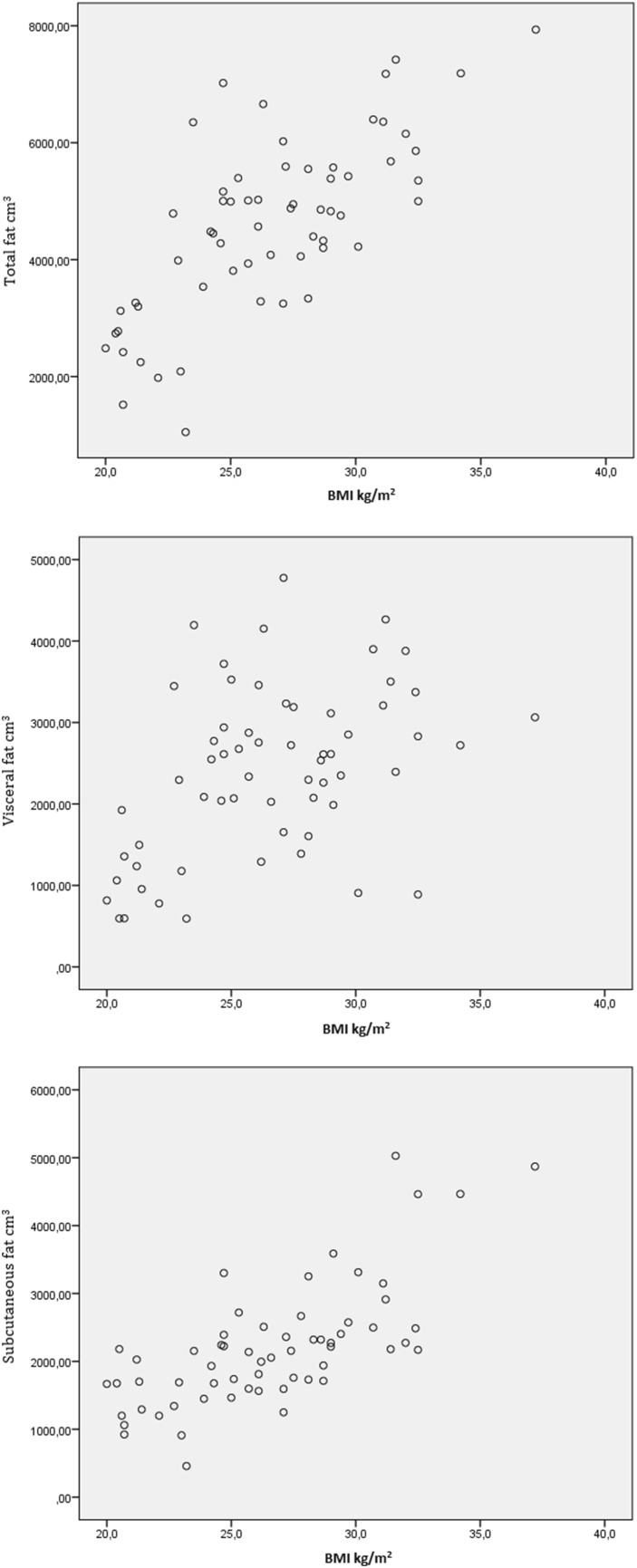
Scatterplot of BMI versus different fat volumes demonstrating correlations of *p* = 0.73, *p* = 0.44 and *p* = 0.75 for total fat versus BMI, visceral fat volume versus BMI and subcutaneous fat volume versus BMI, respectively

Currently, BMI is used in clinical practice to predict adverse outcomes after hernia repair. However, in our population VFV was a significant predictor of reherniation whereas BMI was not. This may be explained by the poor correlation between BMI and visceral fat (Fig. 4). In addition, a recent study found that BMI only increased the risk of reherniation when BMI was over 30 kg/m^2^ [13]. Therefore, visceral fat mass may more accurately predict reherniation than BMI.

SFV was significantly associated with a lower incidence of SSI in our study population. Recently, Levi et al. described subcutaneous fat as a risk factor for infection in patients who underwent CST for ventral hernia repair [14]. However, the BMI in our population (26.5 kg/m^2^) is substantially lower than in Levi’s (33 kg/m^2^). Since BMI and SFV seem strongly correlated (*p* = 0.75 in the current study), the possible negative effects of subcutaneous fat on SSI might only become apparent above a certain SFV [15]. Further prospective research could clarify the role of subcutaneous fat for predicting SSI in both obese and non-obese populations.

A major advantage of our study over others is the accuracy in which the fat volumes were determined. Using a volume calculated of multiple CT coupes (in contrast to using a single coupe), and correcting each measurement manually there is a high degree of construct validity [14]. However, this approach was quite time consuming and we are currently working on a proxy that can be measured easily and fast to make it more readily applicable in practice.

A statistical limitation of our study is the relatively small sample size. Our inclusion number is lower than expected, mainly because a CT scan was not available prior to surgery in all patients. Moreover, throughout the years, CT scan protocols had been modified. Therefore, in certain periods, some (most upper and lower) parts of the abdomen were not scanned and these patients had to be excluded from this retrospective analysis.

## Conclusion

Our study indicates that visceral fat volume, subcutaneous fat volume and hernia sac volume derived from CT scan measurements may be used to predict reherniation and SSI in patients undergoing complex ventral hernia repair using CST. With this information, prospective trials may further identify the role of these CT scan-derived body morphometrics for patient-tailored risk assessment. Our findings need confirmation in future prospective studies, preferably multi-centered to allow for greater study sizes and analysis of more homogenous groups.
